# NETfacts: An integrated intervention at the individual and collective level to treat communities affected by organized violence

**DOI:** 10.1073/pnas.2204698119

**Published:** 2022-10-28

**Authors:** Katy Robjant, Sabine Schmitt, Samuel Carleial, Thomas Elbert, Liliana Abreu, Amani Chibashimba, Harald Hinkel, Anke Hoeffler, Anja C. Rukundo Zeller, Brigitte Rockstroh, Anke Koebach

**Affiliations:** ^a^Nongovernment organization vivo international e.V., 78430 Konstanz, Germany;; ^b^Department of Psychology, Clinical and Neuropsychology, University of Konstanz, 78457 Konstanz, Germany;; ^c^Department of Politics and Administration, Development, University of Konstanz, 78457 Konstanz, Germany;; ^d^Private address, Kigali, Rwanda

**Keywords:** rape myth, intervention, mental health, conflict, sexual violence

## Abstract

War and crises affect mental health, social attitudes, and cultural norms, which can exacerbate the state of long-term insecurity. With decades of armed conflict, the Democratic Republic of Congo is one example, and violence has become normalized in civilian settings. In this study, we tested the effectiveness of the NETfacts health system, an integrated model of evidence-based individual trauma treatment (Narrative Exposure Therapy [NET]) and a trauma-informed community-based intervention (NETfacts). Alongside changes in mental health outcomes (posttraumatic stress disorder, depression, social disapproval, and shame) we also investigated change in attitudes, including rape myth acceptance, stigmatization of survivors of sexual violence, and skepticism about the reintegration of former combatants. To test whether the additional community intervention is superior to individual NET alone, we implemented a randomized controlled design with six villages and interviewed a sample of 1,066 community members. Our results demonstrate that the NETfacts health system in comparison with NET alone more effectively reduced rape myth acceptance and with it ongoing victimization and perpetration. Community members of the NETfacts group also presented with less stigmatizing attitudes against survivors of sexual violence. Skepticism about the reintegration of former combatants declined in both groups. NETfacts appears to have increased motivation to engage in individual treatment. Synergizing the healing effects of individual and collective trauma exposure, the NETfacts health system appears to be an effective and scalable approach to correct degrading or ignominious norms and restore functioning and mental health in postconflict communities.

## Significance

War and crises cause tremendous suffering and hardship and contribute to wider problems in our globalized world (e.g., mass migration, modern slavery, poverty). Yet theoretically anchored, evidence-based tools to mitigate the negative effects and restore resilience in affected communities have remained scarce, despite costly international programs. This article presents a longitudinal trial building on evidence from psychological trauma treatment and peacebuilding. Using trauma-focused individual treatment combined with a community-based intervention, we show that ignominious social norms and attitudes can be addressed effectively with the community-based intervention and thus indirectly reduce ongoing violence.

Like many postconflict zones, the Eastern Democratic Republic of Congo (DRC) continues to be plagued by ongoing violence and unrest. Despite years of investment in peacekeeping and multiple peace agreements, armed groups continue to operate (1), rendering civilians vulnerable to unpredictable violent attacks. Rates of gender-based violence in Eastern DRC are high (2). Across global conflict areas, sexual violence affects all members of the population (men, women, and children), and is perpetrated not only by combatants but also by civilians, in communities and in the home (3).

In a review of studies involving survivors of sexual violence in conflict areas, including 12 studies in DRC, Ba and Bhopal (4) found high prevalence rates of mental health problems. In three related studies in the region, we found high rates of both gender-based violence and perpetration of extreme violent acts and related mental health problems among men, women, and children who had been in armed groups (5–7). In all studies, symptoms of posttraumatic stress disorder (PTSD) and elevated aggression were prevalent among traumatized perpetrators, consistent with findings in other conflict and postconflict settings (8–15). This is also consistent with findings in Western settings that living in a highly aggressive environment can promote the adoption of aggressive behavior (16).

According to Elbert et al. (17, 18) the concept of appetitive aggression can be useful in understanding the dynamic between violence perpetration and victimization and the cooccurring psychological sequelae of both PTSD and aggression, leading to ongoing perpetration of violence in the community once the conflict is over. Appetitive aggression, distinct from reactive aggression in neural circuitry (19–21), is the phenomenon whereby individuals who have perpetrated violent acts are drawn toward recalling, planning, witnessing, and continuing to perpetrate acts of aggression for personal gratification and satisfaction (18). A “bi”-cycle of violence has been proposed whereby both PTSD symptoms and appetitive aggression independently motivate ongoing violence perpetration among traumatized individuals (17). On one side, the sequelae of trauma and the experience of violence with PTSD, depression, and dissociation lead to more impulsivity and reactive aggressive behavior. Similarly, on the other side perpetration (forced or voluntary) increases appetitive aggression and psychopathy, leading to proactive violence. As a result of the relationship between experience of interpersonal violence as victims and aggressivity (2, 22, 23) and also the absence of a functioning legal and peacekeeping capability in Eastern DRC, the reality is that the distinction between victim and perpetrator is false, with many people having been both victims and perpetrators of violence (5, 6).

The responses of others (in the family and in the community) can also influence the mental health sequelae of trauma and perpetration of violence. Social attitudes, including stigma and marginalization of survivors (including perpetrators), exacerbate the mental health problems caused by the events themselves (7, 24, 25), with negative impacts on individual and collective functioning.

Among traumatized communities, stigmatizing views and the exclusion and marginalization of others may hold a specific purpose in contexts where violence is continuous in that it helps to maintain a sense of safety in an objectively insecure setting; Koebach and Robjant (26) provide a detailed theoretical model. In short, research from social sciences has long supported the theory that attitudes and beliefs that emphasize the characteristics of victims as the reason for victimhood serve the function of alleviating anxiety in preference for a restored belief in a just world, as long as certain agreed norms are adhered to (27–30). The difficulty in the current context is that the violence is ongoing, resulting in a marked cognitive dissonance that may further reinforce the victim-blaming attitudes through confirmation bias (31) and social influence (32). These render the victim blaming and derogation congruent characteristics more salient and allow counterfactual narratives to emerge. At the community level there is therefore a collective avoidance that mirrors the avoidance seen at the individual level in the trauma survivor suffering from PTSD. In this way, just as at the individual level, we propose collective avoidance as a key mechanism to serve the function of providing a perceived protection from negative emotions, negating the need for action to prevent further harm, in the face of learned helplessness caused by the frequency of unpredictable attacks (26). Consequently, the individual experiences of traumatization and perpetration are never heard, both because of individual traumatic silence and also because of the reinforcement of this avoidance at the collective level. The counterfactual narratives, negation of individual suffering, and blaming attitudes further result in the withdrawal of caregiving and support to survivors and the absence of proactive action to safeguard the community against future perpetration. In our previous work, we found high levels of rape myth acceptance, stigmatization, and skepticism against the former combatants to deteriorate mental health and peace (7, 24).

## The NETfacts Health System: Treating Trauma and Violence at the Individual and Collective Levels

**Fig. 1.**
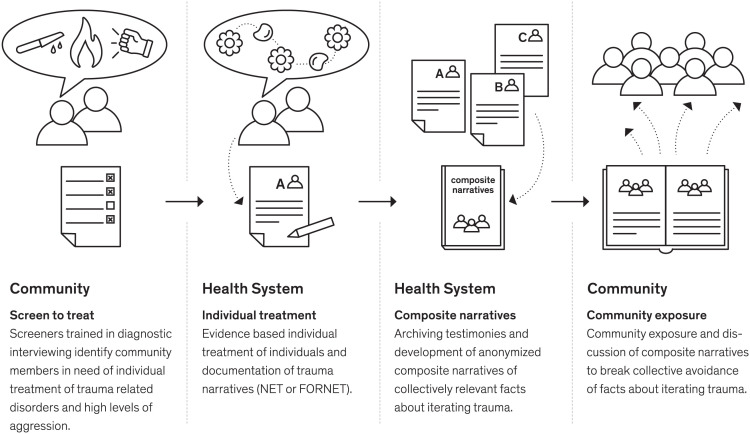
Principles of the NETfacts health system.

Given the emerging evidence that exposure to both victimization and perpetration of interpersonal violence leads to mental health sequelae including PTSD and aggression, and with it (on the behavioral level) ongoing violent perpetration, we propose that both issues must be treated simultaneously at the individual level (e.g., 5, 6). However, considering the additional impact of social attitudes and stigmatizing attitudes and behaviors and the collective avoidance we theorize to be occurring at the community level, we consider that the community itself becomes impaired to effectively care for and safeguard the individuals within it, and therefore a community intervention would be additionally beneficial. We have therefore developed the NETfacts health system (Fig. 1), which attends to individual needs and also provides a community intervention (33).

In our NETfacts health system model for DRC, we screened all individuals within specified communities for either PTSD or significant aggression and offered evidence-based treatment to these individuals via local counselors. The treatments were narrative exposure therapy (NET) (34) or narrative exposure therapy for forensic offender rehabilitation (FORNET, hereafter referred to as NET), two treatments that have been effectively used in the region previously (5, 6, 35) and have a solid evidence base for the treatment of trauma spectrum disorders in survivors of multiple trauma [for review see (36)]. In addition to the narrations of traumatic experiences, NET includes the “lifeline,” a procedure whereby survivors lay out in chronological order their most arousing experiences, using symbols such as flowers (for positive experiences) and stones (for traumatic stressors; see Fig. 1), as well as sticks (for perpetration) in cases of FORNET. All individuals who needed individual treatment were offered this. We then offered four community intervention sessions to all members of the NETfacts communities. In the initial session the community laid out a community “lifeline” whereby a consensus was gathered about the main events that had been important and arousing for the community as a whole. Different individual experiences that occurred within each event were deliberately discussed as a first step to illuminating differing narratives held by individuals who had all experienced the same event but in different ways (e.g., a “village attack” for some meant abduction into an armed group, for others it meant experience of rape, for some it meant witnessing the murder of loved ones). Individuals were also invited to provide a brief account of their own experience as part of the collective memory of the community if they wished to. In the following 2 weeks, local counselors then offered all community members the opportunity to process such an experiences following the technique of NET (aside from those already in a full individual treatment); subclinical community members may benefit from processing individual events according to the understanding of the cumulative impact of trauma on mental health and the building block theory (37). In the final three sessions, trauma counselors then selected the most relevant events (according to the community lifeline and the content of the individual sessions held in the preceding 2 weeks) and facilitated sessions whereby anonymized composite narratives were delivered [derived from individual treatments conducted during simultaneous projects; see Robjant et al. (33) for details]. Facilitated discussions were held regarding the impact of the narrative, the needs of the protagonists in the narrative, the needs of the community, and practical action plans to support reintegration and recovery. As a final step, a commemoration ceremony was held and a depiction of the community lifeline was passed over to the community. Following pilot testing in our feasibility trial (38, 39), we envisage that the gradual breakdown of collective avoidance already begins in the lifeline stage, and especially with the expansion of the perception of collective events to include multiple individual narratives. In the 2-week break where individual events are explored, avoidance is further challenged at the individual level, and finally, in the community sessions through the facilitated discussion, community members are exposed to the emotional details of experiences shared by others and also a facilitated discussion (together with practical actions) about the recourse needed to integrate and support survivors (including those who may have perpetrated violence).

With the present randomized controlled trial study, we aimed to evaluate the effectiveness of a community-based trauma therapy intervention (NETfacts health system; hereafter NETfacts) over an established individual-based trauma therapy intervention (hereafter NET only) in the Eastern DRC. To this end, we interviewed a representative sample of participants (*n* = 1,066) in six communities, which were randomly assigned to one of the two interventions. Follow-up interviews were conducted after NETfacts intervention in a posttest and after 3 and 6 mo. In the NET only intervention, individuals initially waited for treatment start (from baseline to posttest) but were also followed up after 3 and 6 mo. We opted for a high accuracy of outcome estimation (especially mental health) in each community at the cost of potential cluster effects due to the risk of mental health deterioration associated with truth telling and debriefing interventions shown in large longitudinal studies (40–42). We hypothesized that engagement in individual treatment is higher in the NETfacts than in the NET only condition as testimony is encouraged for all community members. The exposure to composite trauma narratives may trigger aversive memories in traumatized community members but also facilitates social support. Therefore, we investigated positive and negative effects in mental health outcomes (PTSD, depression, general disapproval, shame) that may have emerged after the implementation of NETfacts (H2a–d). Regarding social outcomes, we hypothesized superiority in NETfacts communities compared to NET only in rape myth acceptance, stigmatizing attitudes toward survivors of sexual violence, and skepticism about the possibility of reintegration of former combatants. Improvements on these outcomes are further hypothesized to mediate the effect of treatment upon ongoing violence (victimization and perpetration). Finally, a qualitative investigation aimed to complement the above-mentioned analyses by describing the most significant changes triggered by NETfacts according to community member narratives.

## Results

### Study Participants and Baseline Characteristics.

**Fig. 2.**
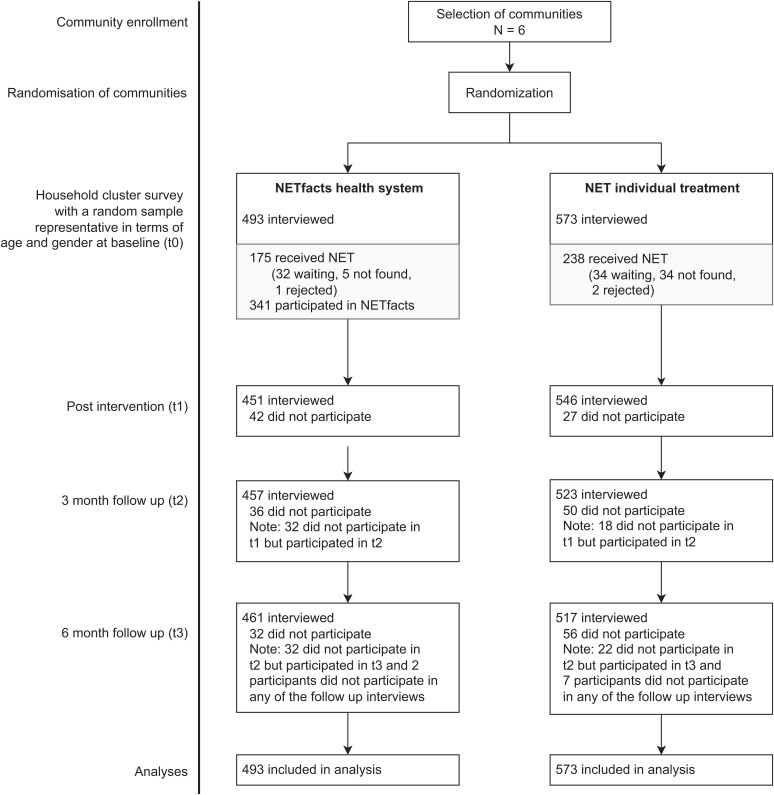
Flow of participants.

Fig. 2 and Table 1 depict the flow of participants. Table 2 presents sociodemographic data of the participants; individuals in the two conditions did not differ in any of these characteristics. The attrition rates for participants lost for all follow-up were minimal (<1%). Individuals with higher education more frequently did not attend the follow-up interviews, but this did not vary across the groups, and none of the other sociodemographic or outcome variables were associated with partial attrition (for details see *SI Appendix*, 1).

**Table 1.** List and flow of activities in the NETfacts and NET only conditions

Activity	NETfacts	NET only	Weeks
Baseline interviews	x	x	1–4
Referral to NET and FORNET	x	x	4f
Community lifeline session	x		5
Provision of singular individual sessions	x		6 and 7
Community exposure sessions 1–3	x		8 and 9
Posttests, 3 and 6 mo follow up, and qualitative interviews	x	x	10f
Delivery of NET and FORNET	x	x	10f

NET = Narrative Exposure Therapy; FORNET = Narrative Exposure Therapy for Forensic Offender Rehabilitation; f = following; mo = months.

**Table 2.** Sociodemographic characteristics

		NETfacts	NET only	Test statistic
Sample size	*n*	493	573	
Age (y)	Mean (*SD*)	36.5 (17)	36.1 (16.3)	*t*_(1027)_ = 0.4
Sex (female)	*n* (%)	252 (51%)	301 (53%)	χ^2^_(1)_ = 0.16
Migrated to community	*n* (%)	265 (54%)	285 (50%)	χ^2^_(1)_ = 1.55
Education (y)	Mean (*SD*)	5.5 (4.4)	5.1 (4.6)	*t*_(1,051)_ = 1.35
Relationship				χ^2^_(2)_ = 2.08
Single	*n* (%)	147 (30%)	150 (26%)	
Married	*n* (%)	298 (60%)	358 (62.5%)	
Widowed	*n* (%)	48 (10%)	65 (11%)	
Children (no.)	Mean (*SD*)	3.8 (3.6)	4 (3.5)	*t*_(1,032)_ = −0.71
GBV experience	*n* (%)	114 (23.1%)	154 (27%)	χ^2^_(1)_ = 1.79
Military participation	*n* (%)	69 (14%)	68 (12%)	χ^2^_(1)_ = 0.89

***P < 0.001; **P < 0.01; *P < 0.05; °*P* < 0.1.

### Effectiveness of NETfacts.

Compared to the NET only group, NETfacts led to an increased reduction in acceptance of rape myth and an associated reduction in actual victimization and perpetration as well as an immediate reduction in PTSD and increased likelihood of engagement in NET subsequently. Descriptive statistics for study outcomes are provided in Table 3. Between-group comparisons did not reveal significant differences at baseline. For details on community baseline differences and the account for cluster effects, see *SI Appendix*, 2. For prevalence rates of PTSD and major depression diagnosis across the three time points, see *SI Appendix*, 3.

**Table 3.** Descriptive statistics of outcomes for mental health and negative social attitudes and norms

		Baseline	Posttest	3-mo follow-up	6-mo follow-up
*Posttraumatic stress symptom severity (PSSI sum score) (H2a)*					
NETfacts	Median (range)	5 (0–62)	0 (0–57)^1^	0 (0–48)	0 (0–53)
NET only	Median (range)	6 (0–57)^2^	6 (0–61)	0 (0–53)	0 (0–41)
*Depression symptom severity (PHQ-9 sum score) (H2b)*					
NETfacts	Median (range)	6 (0–27)	5 (0–24)^1^	4 (0–27)	3 (0–25)
NET only	Median (range)	6 (0–26)^2^	6 (0–24)	4 (0–26)	3 (0–21)
*General disapproval (SAQ subscale sum score) (H2c)*					
NETfacts	Mean (*SD*)	5.1 (3.9)	4.4 (3.8)	4.8 (4.1)	3.2 (3.7)
NET only	Mean (*SD*)	5.4 (3.9)	4.7 (3.9)	4.6 (4.1)	3.0 (3.6)
*Shame (SVQ sum score) (H2d)*					
NETfacts	Mean (*SD*)	29.8 (11.3)	27.5 (12.3)	30.7 (12.2)	29.9 (12.3)
NET only	Mean (*SD*)	29.8 (11.5)	27.9 (12.2)	30.5 (12.1)	29.9 (12.6)
*Rape myth acceptance (IRMA sum score) (H3)*					
NETfacts	Mean (*SD*)	29.1 (7.8)	26.3 (6.9)	27.3 (6.9)	26.0 (7.4)
NET only	Mean (*SD*)	29.6 (7.2)	29.4 (6.7)	28.0 (6.5)	27.8 (6.5)
*Strongly disagreed that survivors of SV have gotten what they deserve when they get stigmatized (ATSS) (H4)*					
NETfacts	*n* (%)	336 (68%)	356 (79%)	355 (78%)	375 (82%)
NET only	*n* (%)	385 (67%)	372 (68%)	377 (72%)	392 (76%)
*Strongly disagreed that when SV happened to a family member, it should remain secret (ATSS) (H4)*					
NETfacts	*n* (%)	193 (39%)	110 (24%)	135 (26%)	121 (26%)
NET only	*n* (%)	250 (44%)	158 (29%)	117 (26%)	143 (28%)
*Strongly disagree that survivors of SV should feel ashamed for what they have done (ATSS)*					
NETfacts	*n* (%)	96 (20%)	83 (18%)	80 (18%)	111 (24%)
NET only	*n* (%)	101 (18%)	104 (19%)	90 (17%)	110 (21%)
*Strongly agreed to take care of a family member who was experiencing trouble due to SV (ATSS) (H4)*					
NETfacts	*n* (%)	373 (76%)	387 (86%)	395 (86%)	409 (89%)
NET only	*n* (%)	439 (77%)	438 (80%)	417 (80%)	435 (84%)
*Skepticism against the reintegration of former combatants (SORS sum score) (H5)*					
NETfacts	*n* (%)	32.1 (10.9)	27.7 (10.0)	27.1 (9.4)	26.2 (10.4)
NET only	*n* (%)	32.9 (10.9)	30.8 (10.3)	27.3 (9.6)	26.6 (10.2)

Note: PSSI = Posttraumatic Stress Symptom scale according to DSM-5–interview version (43); PHQ = Patient Health Questionnaire–9 (44); SAQ = Social Acknowledgment Questionnaire (45); SVQ = Shame Variability Questionnaire (46); IRMA = Illinois Rape Myth Acceptance scale (47); ATSS = Attitudes Toward Survivors Scale (48); SV = sexual violence; SORS = Social Reconstruction Scale (49).

^1^*n* = 572.

^2^*n* = 450.

#### Mental health (H1, H2a–d).

A total of 195 (39.5%) interviewed community members in the NETfacts and 272 (47.5%) in the NET only communities were referred to individual treatment. Engagement was high in both groups: Only 6 out of 213 individuals did not start or finish treatment in the NETfacts condition and 34 of 308 in the NET only condition. According to our hypothesis, we found a greater likelihood of engagement in individual treatments in the NETfacts condition compared to NET only (χ^2^_[1]_ = 9.4, *P* = 0.002). Generalized linear mixed models (GLMMs) further revealed a significant time × group interaction effect from baseline to posttest in participants who were diagnosed with PTSD and referred for, but had not yet received, individual treatment (NET) for PTSD (Likelihood Ration Test [LRT]; χ^2^_[3]_ = 11.9, *P* = 0.008) and depression (LRT; χ^2^_[3]_ = 10.1, *P* = 0.014). Post hoc analysis revealed a decline in PTSD (*t*_[Tukey]_ = 4.6, *P* < 0.001) and depression (*t*_[Tukey]_ = 3.8, *P* = 0.005) from baseline to posttests in the NETfacts condition but not in NET only, indicating that NETfacts could be used as a group intervention to reduce PTSD (H2a) and depression symptoms (H2b). Interaction effects for general disapproval (H2c) and shame (H2d) did not reach significance (for details see *SI Appendix*, 4). Testing for positive or negative effects of NETfacts in the whole sample, the number of individuals who presented with a reliable symptom improvement or deterioration in PTSD did not differ between conditions. Regarding depression, more individuals deteriorated in the NET only condition than in NETfacts (χ^2^_[1]_ = 4.5, *P* = 0.036). Individuals who improved did not differ between conditions (for more details see *SI Appendix*, 4). GLMMs did not reach significance for the other mental health outcomes (depression, general disapproval, shame; for details see *SI Appendix*, 5).

#### Rape myth acceptance.

**Fig. 3.**
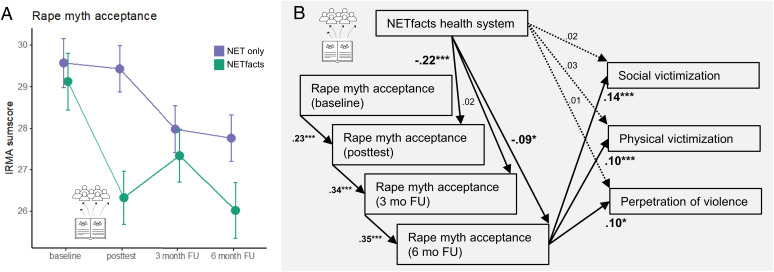
(*A*) Variation of rape myth acceptance in the NETfacts (green) and NET only (blue) conditions across four assessment points. (*B*) Structural equation model diagram showing the direct and indirect effects of treatment intervention and rape myth on current victimization and perpetration. Note: (*A*) IRMA = Illinois Rape Myth Acceptance scale (47); NET = narrative exposure therapy (34); NETfacts = facts derived from NET (26, 33). Error bars represent CIs. NETfacts community meetings took place between baseline and posttests. Individual treatments started after the posttests. (*B*) Path model analyses model fit: robust χ^2^ = 91.5, *P* < 0.001, CFI = 0.94, RMSEA = 0.07, SRMR = 0.04.

GLMM revealed a significant time × group interaction effect (LRT; χ^2^_[3]_ = 31.0, *P* < 0.001; Fig. 3A) showing that NETfacts reduced the acceptance of rape myths more strongly than NET only. Importantly, post hoc analysis showed significant contrasts between the groups at posttests (*t*_[Tukey]_ = 6.7, *P* < 0.001) and 6-mo follow-up (*t*_[Tukey]_ = 3.2, *P* = 0.033). On average, higher levels of rape myth acceptance were associated with older age (LRT; χ^2^_[1]_ = 20.0, *P* < 0.001) and less education (LRT; χ^2^_[1]_ = 60.4, *P* < 0.001; for details see *SI Appendix*, 6).

#### Stigmatization.

A significant effect of the interaction term was found for the number of individuals who strongly disagreed that survivors of sexual violence deserved to be socially excluded (item 1; LRT; χ^2^_[3]_ = 8.2, *P* = 0.042). Post hoc testing indicated that this changed particularly after the NETfacts community meetings, revealing significantly higher disagreement with this statement in the NETfacts group at posttest (*z* = –3.7, *P* = 0.006; OR = 0.5) as well as an increase from baseline to posttest in the NETfacts condition (*z* = –3.5, *P* = 0.012; OR = 0.5) but not in the NET only condition. Older age (LRT; χ^2^_[1]_ = 55.8, *P* < 0.001) and higher education (χ^2^_[1]_ = 26.7, *P* < 0.001) were associated with lower rates of stigmatization in this item. For those who strongly disagreed that incidents of sexual violence against a family member should remain a secret, we also found a significant interaction (item 2; LRT; χ^2^_[3]_ = 9.4, *P* = 0.024), but post hoc tests did not indicate significant differences between the groups at the follow-up time points. Younger age was correlated with higher rates of agreement that sexual violence should remain a secret (LRT; χ^2^_[1]_ = 4.7, *P* = 0.030). We did not find significant differences between the two groups in regard to strong disapproval for the statement “I believe that survivors of sexual violence should feel ashamed for what happened.” Moreover, we found a significant interaction for the number of individuals who strongly agreed that they would help a relative who encountered problems due to sexual violence (LRT; χ^2^_[3]_ = 8.9, *P* < 0.031). Although between-group contrasts did not reach significance, odds ratios were almost double in the NETfacts condition (odds ratio_NETfacts_ = 0.6 and odds ratio_NETonly_ = 0.3 from baseline to 6-mo follow-up), indicating stronger support in the NETfacts condition. Notably, gender based violence (GBV) survivors (LRT; χ^2^_[1]_ = 4.1, *P* = 0.042) and male (LRT; χ^2^_[1]_ = 5.6, *P* = 0.019), older (LRT; χ^2^_[1]_ = 8.3, *P* = 0.004), and more educated participants (LRT; χ^2^_[1]_ = 7.0, *P* = 0.008) presented with higher approval rates (for full model details see *SI Appendix*, 7).

#### Skepticism against the reintegration of former combatants.

GLMM showed a significant interaction effect of time × group (LRT; χ^2^_[3]_ = 20.7, *P* < 0.001). Indicating a lower level of skepticism against the reintegration of former combatants in NETfacts compared to NET only, post hoc tests showed a significant difference between groups at posttest (*z*_[Kenward–Roger]_ = 4.3, *P* < 0.001). On average, higher levels of skepticism against the reintegration of former combatants were associated with female sex (χ^2^_[1]_ = 35.6, *P* < 0.001), less education (χ^2^_[1]_ = 27.6, *P* < 0.001), and nonmigration status (χ^2^_[1]_ = 6.0, *P* = 0.014; for full model details see *SI Appendix*, 8).

#### Victimization and perpetration.

While results did not show evidence for a direct effect of treatment on victimization and perpetration, the mediation effect of treatment through rape myth acceptance on social (*z* = –2.56, *P* = 0.010) and physical victimization (*z* = –2.2, *P* = 0.027) and perpetration (*z* = –6, *P* < 0.001) was significant at 6-mo follow-up. Importantly, rape myth acceptance at 6-mo follow-up positively explained the variation in social (*z* = 4.45, *P* < 0.001) and physical victimization (*z* = 4.74, *P* < 0.001) and perpetration (*z* = 2.18, *P* = 0.029). Thus, path analysis confirmed a significant effect of NETfacts on rape myth acceptance, particularly at posttest (*z* = –4.18, *P* < 0.001) and final follow-up (*z* = –2.21, *P* = 0.027; see Fig. 3). Model fit was acceptable (robust χ^2^_[17]_ = 91.46, *P* < 0.001, CFI = 0.94, RMSEA = 0.07, SRMR = 0.04; for details see *SI Appendix*, 9). Path analysis for stigmatization sum scores and skepticism against the reintegration of former combatants did not reach significance, despite acceptable model fit indices (stigmatization: robust χ^2^_[27]_ = 122.19, *P* < 0.001, CFI = 0.89, RMSEA = 0.06, SRMR = 0.04; skepticism against the reintegration of former combatants: robust χ^2^_[24]_ = 96.75, *P* < 0.001, CFI = 0.94, RMSEA = 0.06, SRMR = 0.03; SI9).

### Qualitative Results.

**Fig. 4.**
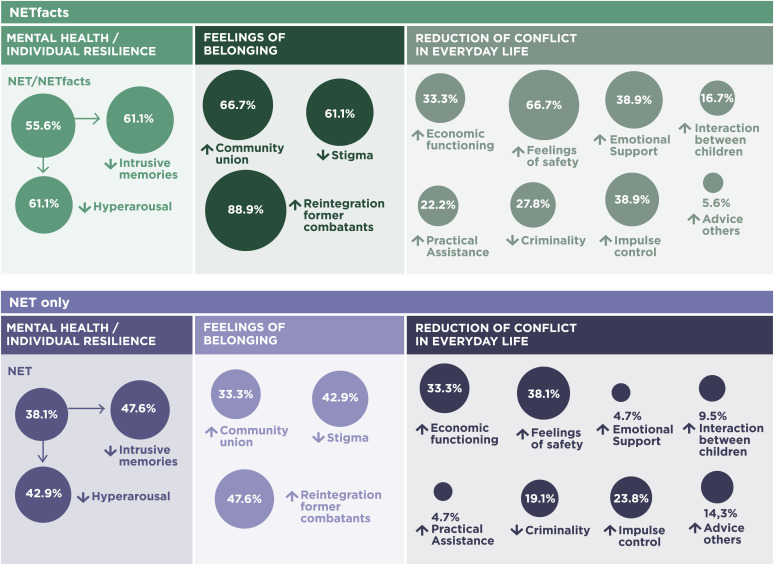
Main themes in the NET only versus NETfacts conditions covering the most significant changes narrated by the participants.

To gain a complementary insight into the changes from the view of the community members, we carried out the first step of the most significant change technique (50–52). The tool aims to participatorily monitor and evaluate the implementation of an intervention including the collection of stories of change, identification of the most significant themes, and discussion of their different values. Results reveal that our predefined outcome measures relate to the changes observed of community members. Participants described actual events and processes in their lives and how these had been interpreted. Three main themes emerged: improvement in mental health, increased feelings of belonging, and a reduction of conflict (including violence and aggression) in everyday life. Each theme includes three to five topics and has different interactions at the individual and collective as well as the group level. To explore potential differences between the two groups, we have analyzed the interviews regarding coherence and frequency of reported changes. While the themes overlapped in both groups, we found more detailed and pronounced changes for all three themes in community members who participated in NETfacts (Fig. 4). For instance, for the theme feeling of belonging, one of the participants described the change as follows: “So, vivo [our implementing partner] helped us, to go close to those people and as community members, as people belonging to the same community, we started living together, sharing sufferings together. We no longer reject them [former combatants/survivors of sexual violence]. We no longer set them away within the community. So, we become, we are united, we’ve become one. So, we are living as one within this community” (FG3, NETfacts condition; for more details see *SI Appendix*, 10).

## Discussion

This study demonstrated that NETfacts can effectively change negative social attitudes that mediate ongoing violence in communities marked by decades of war and crises. Most importantly, results showed a significantly stronger reduction in rape myth acceptance in the NETfacts intervention including individual and community treatment than in the control condition, where communities received individual treatment only (NET, for individuals who had clinically relevant symptoms). This effect was maintained in the 6-mo follow-ups, and lower levels of rape myth acceptance were associated with reduced levels of actual victimization and perpetration. Moreover, we found that some of the stigmatizing attitudes against survivors of sexual violence declined more strongly in residents of communities allocated to the NETfacts condition; that is, community members were more often willing to provide support to family members who needed assistance after an experience of sexual violence. With regard to the reintegration of former combatants, we found an immediate reduction in skepticism in the NETfacts condition, but mean differences disappeared in the later assessments when individual treatments had started. Similarly, symptoms of PTSD and depression declined more strongly after the community meetings in the NETfacts condition than in the communities who awaited individual treatment but not in the long run, and engagement in individual treatment was higher. This also indicates that NETfacts should be implemented as a group intervention to address trauma-related mental illness through a stepped care model. Qualitative interviews support the NETfacts rationale supporting quantitative results: Members of NETfacts communities provided more evidence for the social integration of survivors of sexual violence and former combatants, and with it a reduction in violent offenses.

Following the NETfacts rationale, composite narratives derived from NET sessions correct counterfactual narratives that have become increasingly relied upon as individual and collective avoidance has become the norm. Fragmented accounts of the collective memory are shared and new meanings discussed between community and family members. The increase in incidence of sexual violence in Eastern DRC has also facilitated a normalization of ignominious norms and social degradation of GBV survivors. The NETfacts health system was particularly effective in challenging the resulting myths and led to a lasting change in attitudes against survivors of sexual violence, which ultimately resulted in decreased victimization and perpetration. In contrast, skepticism against the reintegration of former combatants seemed to decrease when community members received NETfacts or those who presented with PTSD and appetitive aggression received individual treatment. Supported by the qualitative interviews, we propose that two different mechanisms led to the stigmatization of survivors of sexual violence and former combatants: prevalent social degradation based on a (defensive) adoption of counterfactual narratives and fear. When they included questions about the social integrity of survivors of sexual violence, qualitative interviews revealed that community members started to exhibit more prosocial behaviors and refrained from public mockery. The reduction in social exclusion of former combatants was more related to perceived engagement in community life on their part (e.g., starting to pay bills, controlling their impulses, and reducing violent attacks). NETfacts community meetings facilitated this inclusion and the practical problem-solving aspects of community integration through the sharing of narratives, and facilitated conversation supported and accelerated the reduction of skepticism. Reflecting this, in the qualitative interviews participants of the NETfacts condition appeared more able to provide concrete examples of proactive change regarding living with former combatants. However, the results highlight the importance of individual treatments for former combatants and traumatized offenders in general to facilitate social integration and allow reconciliation. To our knowledge there is only the study of Cilliers et al. (40), who tested the effectiveness of a comparable community intervention in a prospective randomized controlled trial in Sierra Leone. The authors found that a reconciliation-oriented truth-telling process (“Fambul Tok”) with community members trained in trauma healing and mediation improved social capital, while mental health deteriorated in communities who received this intervention. In contrast, we found that the community meetings in which trauma counselors presented detailed narratives of violent experiences that emerged both in the community’s “lifeline” and NET sessions neither triggered extreme emotional responses during the sessions nor resulted in a subsequent mental health deterioration. Moreover, this new community-level treatment enhances the original evidence-based individual treatment (H1, H2a, b). The crucial difference between our work and that of Cilliers et al. (40) is the symbiotic nature of a reciprocally evolving evidence-based individual treatment and community-based intervention. Thus, a truth-telling process where the cognitive and affective ordeal is actively illuminated and shared through an empowering process by means of the NET technique could therefore improve mental health, change social attitudes, and decrease actual perpetration and victimization. This opens new avenues for cost-effective psychosocial interventions in postconflict settings.

### Outsight.

In this study, accumulated eligibility of participants was large, and we found substantial variation in PTSD symptoms over time; for example, follow-up interviews from baseline to posttest confirmed the diagnostic status in only 45.1% (111 of 246 participants diagnosed with PTSD at baseline). Earlier studies show that such variation may depend on the number of lifetime traumatic events (53), and individuals with higher symptom loads are less likely to present with a temporary decline to subclinical levels (54, 55). As we found a reduction in PTSD and depression symptoms after the NETfacts community meetings, the threshold for individual treatment could be elevated to render the NETfacts health system more efficient. Moreover, communicative and artistic elements sustainably implemented in the communities could boost the effects of NETfacts. Generalizability of the findings beyond the study context should be high as the theorized mechanisms are valid across culture; notably, in each setting the narratives will be different, and thus NETfacts and its outcomes may be different.

### Limitations.

We opted for high accuracy in estimating mental health and social outcomes at the cost of sampling a small number of communities. Although we gained a profound insight and understanding for each community, we were more likely to have unexpected bias due to incidents affecting specific communities. In communities interviewed at the beginning of the trial, more international staff were on site providing supervision. This may have influenced the answering pattern of social outcomes (see *SI Appendix*, 1). Our observations did not indicate major differences at baseline between the conditions, but a higher number of communities would have allowed a more robust analysis.

## Materials and Methods

### Study Design.

To test the effectiveness of the NETfacts health system in comparison with the individual treatment only (NET), we applied a prospective randomized controlled trial with a combined quantitative and qualitative approach (trial registration no. DRKS00015745) and follow-ups after the intervention (posttest) and 3 and 6 mo later.

### Sampling.

Sample size was determined from two angles: We first conducted an a priori power calculation for the total sample aiming at a minimum power of 80% and testing at a 95% significance level, assuming a small effect. Second, to ensure an accurate estimation of outcomes per community, we calculated the sample size based on a 5% margin of error and 95% CI. We opted for a high accuracy of outcome estimation (especially mental health) in each community at the cost of potential cluster effects due to the risk of mental health deterioration associated with truth-telling interventions shown in large longitudinal studies (40, 41).

### Procedure.

The trial was conducted between October 2018 and December 2019 as part of a larger mental health project implemented in collaboration with the nonprofit organization vivo international. Eligibility of participants was determined by residency in the community and age >15 y. Exclusion criteria to participate in the interview were acute intoxication, acute psychotic symptoms, and signs of cerebro-organic disease. All participants provided a written informed consent (fingerprint in case of illiteracy). The trial was approved by the ethical commission of the University of Konstanz and the Fond Social of the DRC. For study protocol see (39).

### Selection of Communities and Participants.

Seventeen communities were preselected based on accessibility from the regional capital (Goma) and availability of trauma counselors. Three communities were immediately excluded for security reasons. Of the 14 communities we selected six communities of which two were comparable in population size and village facilities (e.g., availability of education and work) to account for confounders but showed a sufficient walking distance in between to reduce spillover effects. To this end, each of the 14 communities was visited to get an overview of these factors before final selection. Within each pair, communities were randomly assigned to the NETfacts or NET only condition via simple cluster randomization. Communities and assessors were not actively informed about the allocation of each community, but the information was also not deliberately concealed.

Based on a presurvey of the population in each community (see *SI Appendix*, 11), we interviewed a representative sample based on sex and age stratified across households. To this end, interviewers went from door to door and randomly selected one to two persons per household by blind drawing of folded papers from an envelope. Papers were prior labeled with the respective sex–age categories (man, woman and 16–36 y, 37–57 y, >57 y) to ensure representativeness according to the presurvey.

### Interventions.

In both groups participants identified to have relevant symptoms were referred to a trained trauma counselor in the region and received evidence-based trauma therapy [NET (34) or FORNET (5, 6)]. Treatments then started about 1–2 mo after the screening (after the posttests). In the NETfacts condition, community-based meetings were implemented as described in the introduction by a team of local and international trauma experts and influential community members.

### Assessment Procedure and Measures.

Clinical interviews were conducted by 20 psychological interviewers from the regional capital Goma and its surrounds who were specifically trained for the purpose of the study and blind to the interviewees’ involvement in activities. The training of interviewers included instructions on obtaining informed consent, the use of assessment measures, empathic interviewing, overview of key principles of scientific studies including the importance of confidentiality, information about the overall project and therapeutic intervention to be offered to individuals who required it, and interviewer self-care (since the interviewers were exposed to hearing about severe traumatic events). The interviewers were fluent in the local language, Kiswahili. Before use, the measures were translated into Kiswahili and back-translated into English or French to check for their accuracy. Discrepancies were resolved through discussions between clinical psychologists and local translators. Following the training, interviewers were closely supervised by clinical psychologists including live observations of interviews. Interviews took between 1.5 and 2.5 h, in a confidential setting within the community. Commitment to participate in the study was high due to enthusiasm for the overall project. Participants received a compensation of 1,000 CDF (∼0.60 USD) and light refreshment for participation in the interview.

The assessment battery included questions about sociodemographic characteristics (age, sex, marital status, years of education, migration), as well as exposure to violence and clinical and social outcome measures.

Social outcomes included the acceptance of rape myths, stigmatization, and skepticism against the reintegration of former combatants. The first was measured with an adapted version of the short Illinois Rape Myths Acceptance Scale (IRMA; H3) (47). The original questionnaire comprised four scales: “Rape is a deviant event,” “He didn’t mean to,” “It wasn’t really rape,” and “She asked for it.” After internal discussions with local psychologists about how rape is misconceived in the local context, the scale “She owed him” (four items) was added to assess the belief that in some circumstances sex is owed to men (e.g., in exchange for goods or within marriage). This is in line with Buller et al. (56), who showed in a systematic review that sex is often expected in exchange for favors, and Tavrow et al. (57), who specifically argued for including it in the assessment of rape myths. The final 15 items were rated from 0 (*disagree strongly*) to 4 (*agree strongly*), including questions such as “If a man wants to marry a woman, it is o.k. to force sex on her” or “A woman who dresses in skimpy clothes should not be surprised if a man tries to force her to have sex.” Higher sum scores indicate stronger acceptance of rape myths (range 0–60). Negative attitudes toward rape survivors and willingness to provide support were assessed with the Attitudes and Beliefs toward Survivors of Sexual Violence Scale (48) developed in Kenya and DRC (H4). Participants indicated agreement with four statements on a 5-point Likert scale from 0 (*disagree strongly*) to 4 (*agree strongly*). In line with the original publication (48), no sum score was calculated due to high heterogeneity of items. The Social Reconstruction Scale (SORS; H5) (49) evaluates the readiness to reconcile with former enemies and was developed to assess openness to social reconstruction between Croats and Serbs in postgenocide Bosnia–Herzegovina. The scale was adapted to measure readiness to reconcile with former combatants in DRC (e.g., “I am not ready to cooperate with ex-combatants even if my community asked me to do so”). Two items were removed from the scale as they were nonapplicable in this context. Nineteen items were rated from 0 (*disagree strongly*) to 4 (*agree strongly*). A sum score was calculated to indicate skepticism against the reconstruction with former combatants (range: 0–76).

Exposure to violence was assessed with a short version of the Threats to Human Life scale (58). The 41-item act-based checklist assesses threats to physical (18 items) and social integrity (8 items) and perpetration of violent acts (15 items) experienced during one’s lifetime and in the last 3 mo. Sum scores were used, with higher scores indicating a higher number of experiences in the category.

Clinical measures included the PTSD Symptom Scale Interview (PSSI; H2a) (43), the Patient Health Questionnaire–9 (PHQ-9; H2b) (44) for depression, the Shame Variability Questionnaire (SVQ; H2c) (46), and the general disapproval subscale of the Social Acknowledgment Questionnaire (SAQ; H2d) (45). The PSS-I and the PHQ-9 instruments allow for an estimation of symptom severity (via sum scores, with higher values indicating more severity) and diagnosis according to the fifth version of the *Diagnostic and Statistical Manual of Mental Disorders* (DSM-5) (59). The instruments have been applied in a variety of cultural settings with high validity and reliability (60, 61), including East Africa (62, 63) and DRC (5, 6, 64). Higher values indicate higher levels of symptom severity. The subscale “general disapproval” of the SAQ was used to assess the perceived lack of social acknowledgment as a trauma survivor. The subscale has shown to be strongly related with PTSD (55, 65, 66) and has been used successfully in previous studies in the region (5, 6). First, participants were asked whether they had ever experienced a traumatic event after which they felt the need for social support. The five items were then rated in reference to this event from 0 (*I do not agree at all*) to 3 (*I completely agree*). Higher values of the sum score indicate that participants perceived a stronger general disapproval for the ordeal that they had survived (range: 0–15). The SVQ was used to measure feelings of shame. Participants referred each of the 14 items to the time when they felt the most shame or worst about themselves in the last 4 mo. Each item ranged from 0 (*not at all/I did not feel this way*) to 4 (*completely/I felt this very strongly*). Items included “I felt I deserved to be punished” or “I wanted to hide from other people,” with two inverse items (“I felt good about myself” and “I felt like a worthy or valuable person”). After the inverse items were recoded, the sum score (range: 0–56) indicated stronger feelings of shame.

### Statistical Analysis.

Descriptive statistics of sociodemographic and outcome variables were calculated for the two intervention groups. χ^2^ and *t* tests were carried out when appropriate to verify between-group differences at baseline and follow-ups. To test our hypotheses regarding the effectiveness of NETfacts in comparison to NET only, we used separate GLMMs with lme4 1.1–27.1 for each response variable (67). Response variables were sum scores of mental (PSSI, PHQ-9, SAQ subscale, and SVQ) and social outcomes (IRMA and SORS). The Attitudes Toward Survivors Scale (ATSS) was also considered as response, but this measure was adopted as single binary items (agree/disagree) and not as a scale (0–4). To test the effect of intervention over time, we added the interaction term time (baseline, posttest, 3- and 6-mo follow-up) × group as a main effect in our models. Control variables were age, sex, years of education, migration status, and GBV experience. Numerical control variables were scaled before model fit, to facilitate interpretation and support model convergence. Individual and interviewer IDs were used as random factors. Models with binary responses were fit to a binomial distribution, whereas the remaining models had a Gaussian error distribution. Significance tests were assessed by likelihood ratio tests (68), and post hoc tests were conducted with emmeans 1.4.6 (69). Finally, to evaluate the longitudinal relationship between treatment and social outcomes on the prevalence of violence, we conducted a path analysis with lavaan 0.6–9 (70). Social outcome sum scores for each of the four assessment times were included as sequential predictors in our model; that is, pretest values (IRMA at baseline) explained posttest values (IRMA at posttest), which explained 3-mo follow-up values, and so on. Final responses were “perpetrated violence” and “social and physical experienced violence.” Treatment condition was set to 0/1 for NETfacts and added as main predictor. Similar to GLMM, controls were also used. Community was set as a nesting effect. We tested the direct and indirect effects of treatment on violence prevalence since the effect of therapy could be assumed to work through measures of outcomes (mediation). Model fit was assessed based on fit indices following Hu and Bentler (71). All statistical analyses were carried out in R 4.0.2 (72).

### Qualitative Assessment and Analysis: Most Significant Change.

#### Assessment.

Thirty-nine semistructured interviews (8–55 min, average 30 min) and 10 group discussions (80–140 min, average 106 min; not included in the diagram) were conducted during the 6-mo follow-up assessments between May and December 2019. Individual interviews and group discussion were purposively sampled to include a wide range of views about the immediate and sustained impact of the NETfacts intervention. Heterogeneity of sampling of individual interviews was used for maximum variation of views and experiences regarding therapy category (FORNET, NETfacts group session, singular exposure session), lifetime trauma (sexual violence, ex-combatants, and other community members), change on stigmatization attitude scales toward sexual violence or armed group survivors since baseline, and demographic characteristics (gender, age, born in the community vs. immigrated). Interviews were conducted by a lead researcher (S.S.) or a Congolese translator, and group discussions were conducted by a lead researcher (S.S.), a Congolese translator, and a comoderator. Group discussions were conducted according to a semistructured discussion guide, lasted 2–3 h, and were audio-recorded, translated from Kiswahili into English, anonymized, transcribed, and stored together with notes made by the moderators during and shortly after the discussions. Participants received light refreshment and financial compensation (8.000 CFC, equivalent to $5).

#### Analysis.

Interview recordings were transcribed verbatim. The intervention process interview was analyzed via thematic analysis (73). Thematic analysis is a flexible approach that provides a rich account and interpretation of the data. The data were analyzed independently by two researchers (including L.A.) according to thematic analysis protocol in NVivo 12 (74) and were merged by consensus following continuous and iterative discussion to strengthen coding consistency. This triangulation was further supported by researchers’ different backgrounds (psychology and public health). In addition, the classifications were always discussed and validated by the last author (A.K.). Open coding, axial coding, and selected coding were used. The quotations with similar meanings were synthesized into categories (open coding), which were then grouped into themes (axial coding), and then into core themes (selective coding). The most illustrative verbatim quotes were selected by two researchers. This process is in line with guidance on conducting a thematic analysis. Comparison of coding by the two independent coders revealed an interrater reliability of 78%. Inductive content analysis (75) was used to analyze general feedback from participants on their experiences and perceptions of NETfacts intervention. Content analysis is a replicable approach to describing and quantifying data that enables new insights and can facilitate the practical application of findings. Following familiarization with the data, concepts were developed from the data, and the frequencies of these between participant interviews were counted.

## Supplementary Material

Supplementary File

## Data Availability

Anonymized quantitative data have been deposited in Open Science Framework (https://osf.io/4x625/) (76).
