# Supplementary material for: NETfacts: An integrated intervention at the individual and collective level to treat communities affected by organized violence
Source: Proc Natl Acad Sci U S A. 2022 Oct 28;119(44):e2204698119. doi: 10.1073/pnas.2204698119 (PMC9636916; doi:10.1073/pnas.2204698119)
Supplement: Supplementary File [file pnas.2204698119.sapp.pdf]

PNAS

Supplementary Information for

NETfacts - an integrated intervention at the individual and collective level for resilient communities: a randomized controlled trial in Eastern DR Congo

Katy Robjant[1], Sabine Schmitt[1], Samuel Carleial[2], Thomas Elbert[1, 2], Liliana Abreu[3], Amani Chibashimba[1], Harald Hinkel, Anke Hoeffler[3], Anja C. Rukundo Zeller[1,2], Brigitte Rockstroh[1], Anke Koebach[1,2,3*]

[1]vivo international e.V., non-governmental organization, Germany

[2]University of Konstanz, Department of Psychology, Clinical and Neuropsychology, Germany

[3]University of Konstanz, Department of Politics and Administration, Development, Germany

*Corresponding author

This PDF file includes:

SI1 to SI11
References
Number of tables: 7
Number of figures: 4

SI1: Attrition

A total of 185 participants that were interviewed at baseline could not be re-interviewed in one of the follow ups but only 9 (2 in the NETfacts and 7 in the NET only condition) were lost for all post and follow up interviews. A total of 176 (17%; $n_{NETfacts}$ = 90; $n_{NET\ only}$ = 86) were re-interviewed but not in all waves. To assess whether attrition might have biased our results, we firstly conducted a correlation analysis of all sociodemographic and outcome variables (see Figure SI1) with attrition. In the next step, we calculated a generalized linear model (GLM) with variables that were significantly associated with attrition according to the correlation analysis. We then tested interaction effects of the variables that were still significant. We found that only higher education was associated with attrition (e.g. they were unable to attend the interview due to obligations at work of travel; *p* = 0.024; see Table SI1), but not specifically with one of the groups (group x education: $Chi^2_{(1)} = 0.94$, $p = 0.332$).

Figure. S1. Pearson correlations for sociodemographic and outcome variables with attrition rates (individual not interviewed in one or more follow ups).

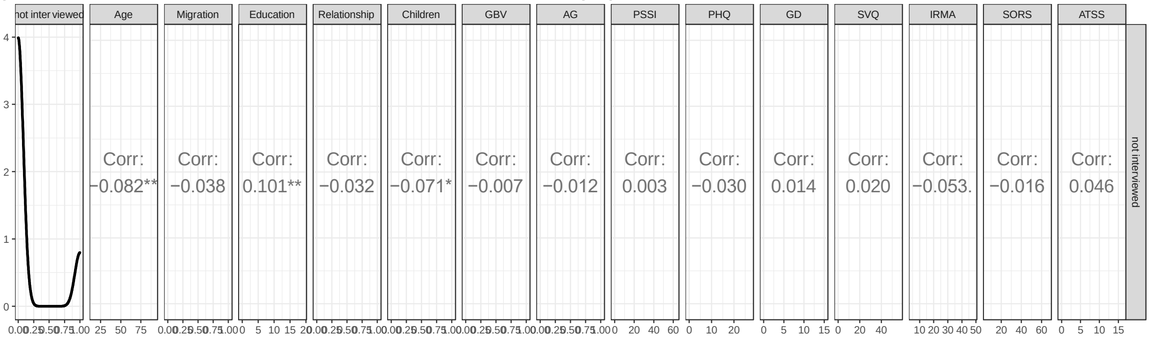

Table S1. Generalized linear model (binomial).

	Estimate	Std. Error	z value	p value	
(Intercept)	-1.60	0.29	-5.48	4.31E-08	***
Group (NET only)	0.21	0.17	1.27	0.2032	
Age	-0.01	0.01	-1.36	0.1737	
Children	-0.08	0.22	-0.38	0.7065	
Education	0.05	0.02	2.26	0.0237	*

Note: Dependent variable: not re-interviewed in one or more follow up interviews.

SI2a: Cluster effects (baseline)

Table S2a. Confidence interval for baseline measures per community (*N* = 6), showing that sociodemographic characteristics at baseline are similar in the NETfacts (*n* = 3; marked with *) and NET only (*n* = 3) condition.

Community	Age	Male	Educ	Migr	GBV	Perp	Vict (s)	Vict (ph)
Ngumba (N = 205)	[35.29-39.86]	[0.44-0.58]	[3.3-4.44]	[0.48-0.62]	[0.18-0.29]	[1.54-2.14]	[3.24-3.78]	[8.55-9.46]
Maoma (N = 217)	[32.8-36.73]	[0.39-0.52]	[5.66-6.94]	[0.44-0.57]	[0.23-0.36]	[2.47-3.13]	[3.7-4.21]	[10.03-10.98]
Kairenge (N = 151)	[33.11-38.92]	[0.38-0.54]	[4.43-5.88]	[0.34-0.5]	[0.21-0.35]	[2.5-3.26]	[4.02-4.7]	[10.04-11.03]
Kihindo* (N = 177)	[35.62-40.74]	[0.42-0.57]	[5.4-6.73]	[0.49-0.63]	[0.13-0.24]	[1.31-1.94]	[3.13-3.71]	[8.4-9.4]
Bukobati* (N = 168)	[33.95-39.08]	[0.42-0.58]	[5-6.32]	[0.39-0.55]	[0.22-0.35]	[2.36-3.12]	[3.53-4.16]	[10.68-11.74]
Katolo* (N = 148)	[31.81-37.23]	[0.39-0.55]	[3.94-5.35]	[0.51-0.67]	[0.16-0.29]	[2.2-2.99]	[3.77-4.4]	[9.38-10.53]

Note: Educ = years of education; Migr = migration into the community; GBV = gender based violence; Perp = number of lifetime acts of perpetration; Vict (s) = number of lifetime experiences of social victimization; Vict (ph) = number of lifetime experiences of physical victimization (threats to physical integrity).

SI2b: Cluster effects (longitudinal)

Figure 1: Mean and standard deviation presented for current social and physical victimization and perpetration at baseline, 3 month follow up and 6 month follow up for each community; green shades represent communities from the NETfacts and violet shades represent communities from the NET only condition.

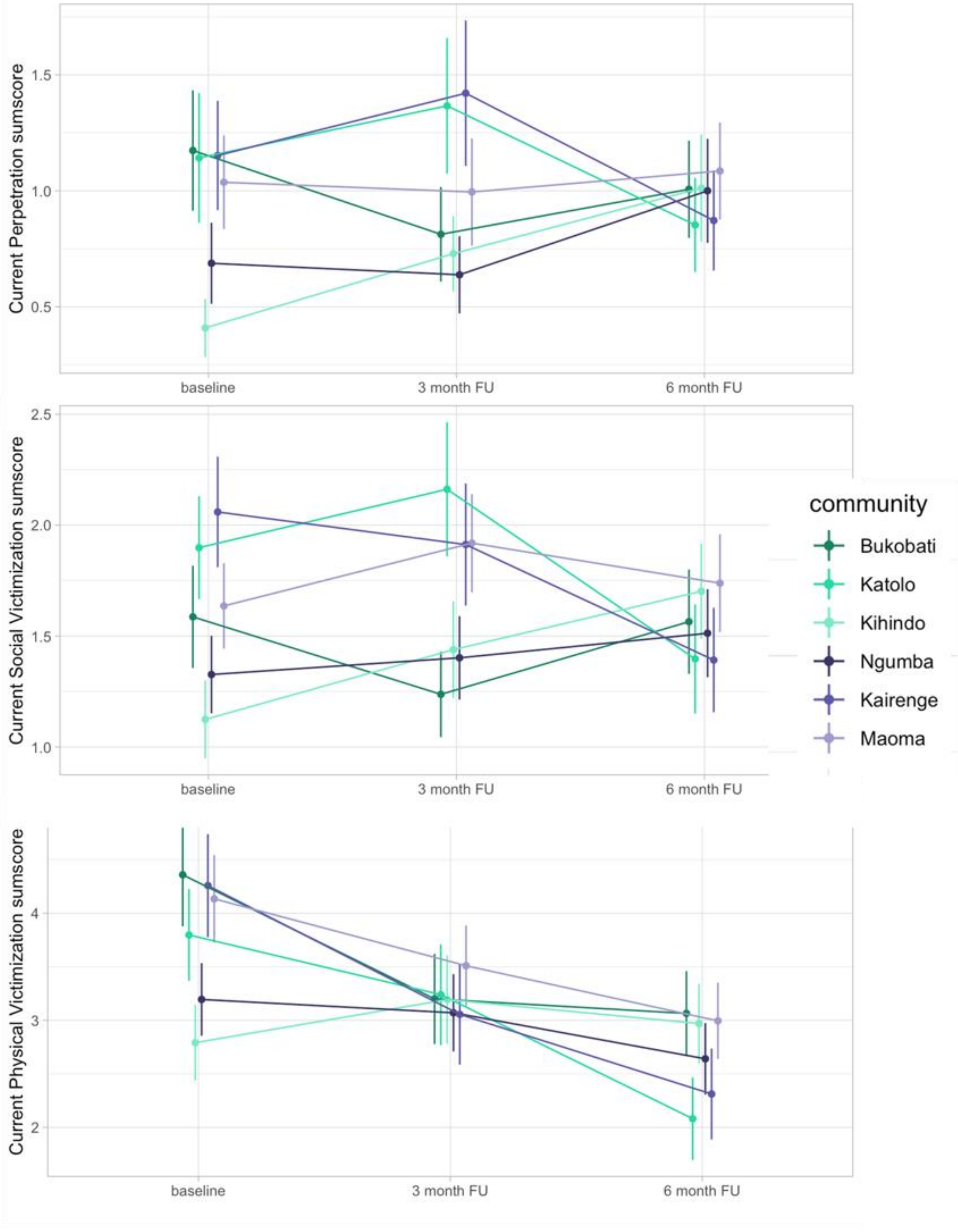

SI2b: Cluster effects (longitudinal; continued)

Figure 2: Mean and standard deviation presented for mental health outcomes at baseline, 3 month follow up and 6 month follow up for each community; green shades represent communities from the NETfacts and violet shades represent communities from the NET only condition.

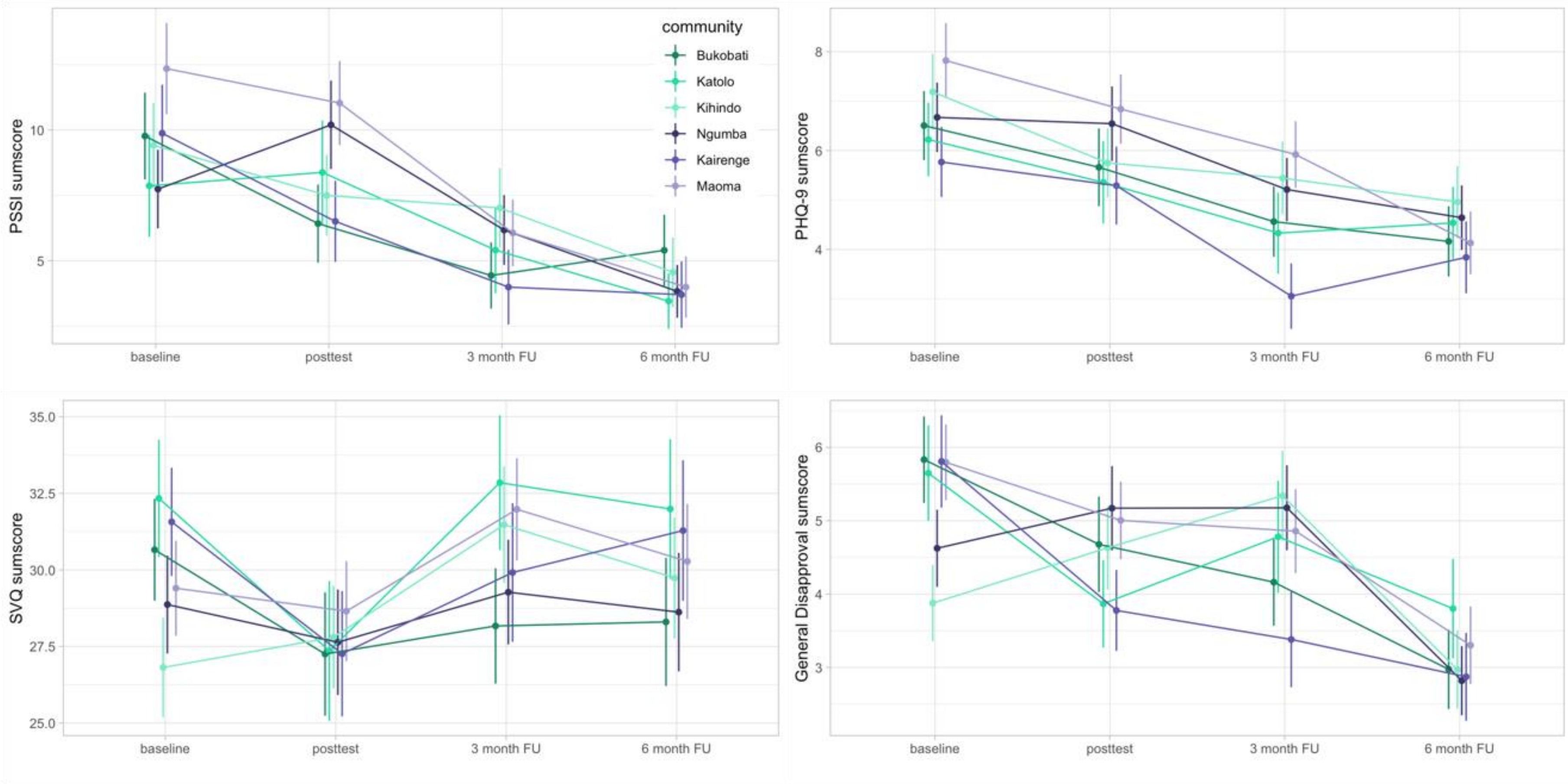

Note: PSS-I = Posttraumatic Stress Symptom Scale – Interview; PHQ-9 = Patient Health Questionnaire 9; SVQ = Shame Variability Questionnaire.

Figure 3: Mean and standard deviation presented for social outcomes at baseline, 3 month follow up and 6 month follow up for each community; green shades represent communities from the NETfacts and violet shades represent communities from the NET only condition.

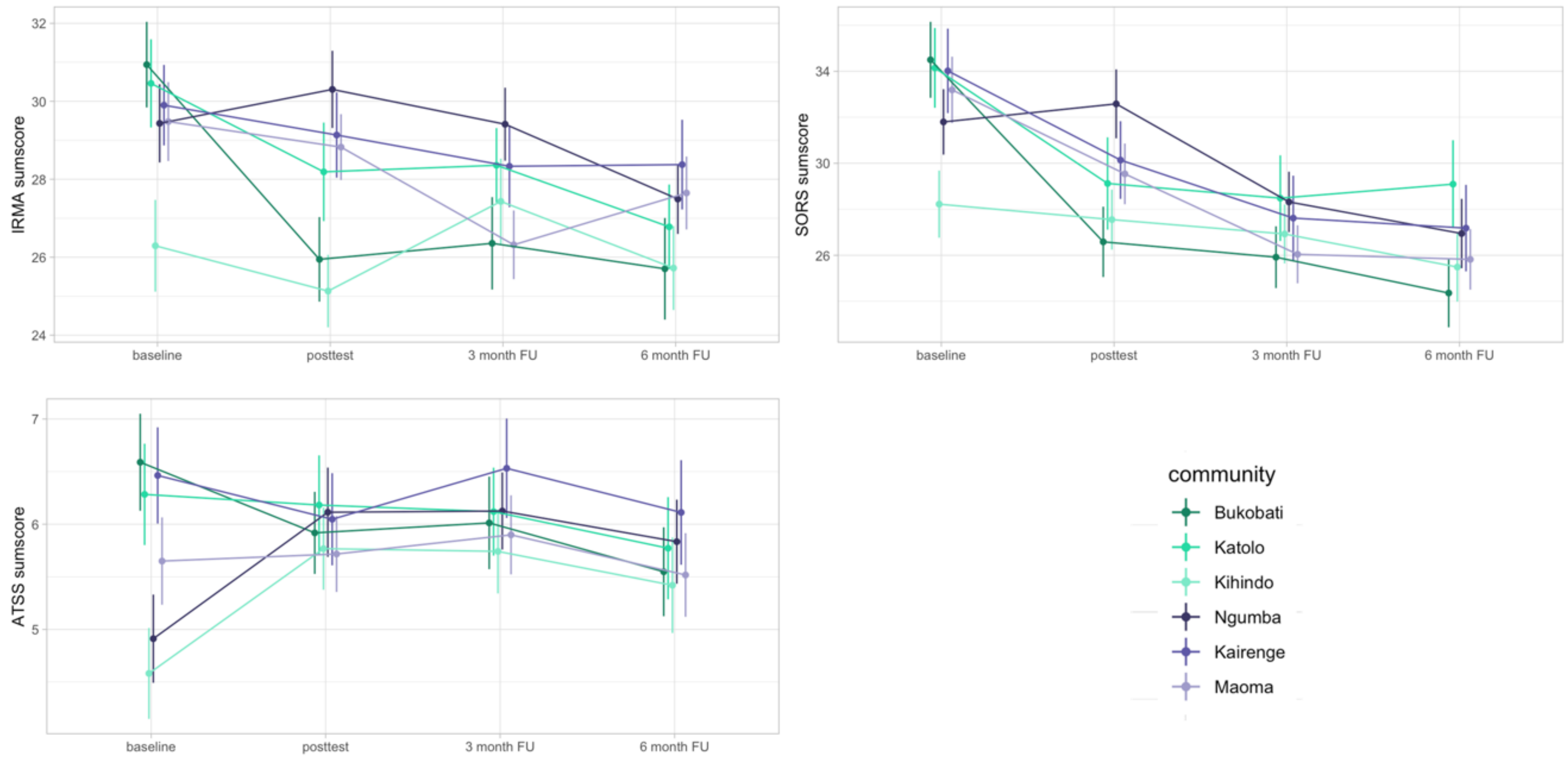

Note: IRMA = Illinois Rape Myth Acceptance scale; SORS = Social Reconstruction Scale; ATSS = Attitudes Towards Survivors Scale.

SI3: Prevalence rates for PTSD and Major Depression diagnosis

Table SI0: Prevalence rates for PTSD and Major Depression diagnosis

	Baseline	NAs	Posttest	NAs	3mo FU	NAs	6mo FU	NAs
PTSD diagnosis								
NETfacts	131 (27%)		105 (23%)	43	90 (20%)	36	74 (16%)	34
NET only	173 (30%)	2	173 (32%)	27	104 (20%)	50	73 (14%)	56
Depression diagnosis								
NETfacts	40 (8%)		26 (6%)	43	27 (6%)	36	19 (4%)	33
NET only	52 (9%)	1	44 (8%)	27	20 (4%)	50	23 (4%)	56

SI4: MH effect of NETfacts community meetings [H2a-d]

Hypothesis: Exposure to traumatic details vs social support has a positive vs negative effect on mental health outcomes (testing PTSD, depression, general disapproval, shame; baseline to posttest).

→ full GLMMS are provided in “DatasetS1.xlsx”

SI5: MH reliable change

Testing for positive or negative effects of NETfacts in comparison to NET only (awaiting individual treatment).

Reliable change PTSD and depression. The number of individuals who presented with a reliable symptom improvement or deterioration did not differ among the conditions (SIx). Reliable change was based on [1, 2](1.96*SD1*sqrt(2)*sqrt(1-rel)) resulting in a required symptom change of ±4.26 for PTSD and ±1.83 for depression.

Table. SI5a. Reliable change indices for PTSD symptoms measure at baseline and posttest (after the NETfacts community meetings)

	NETfacts n = 450	NET only n = 545	Test statistic
no change (n)	334	388	Chi2(1) = 0.99, p = 0.320
deteriorated (n)	116	157	
no change (n)	298	374	Chi2(1) = 0.54, p = 0.461
improved (n)	152	171	

Table. SI5b. Reliable change indices for depression symptoms measure at baseline and posttest (after the NETfacts community meetings)

	NETfacts n = 450	NET only n = 545	Test statistic
no change (n)	326	360	Chi2(1) = 4.41, p = 0.036
deteriorated (n)	124	185	
no change (n)	257	329	Chi2(1) = 0.95, p = 0.330
improved (n)	193	216	

SI6: Rape myth acceptance [H3]

Table S6. Model details of rape myth acceptance.

Statistical model outputs, showing model fit indices, effect estimates and significance tests, followed by *post hoc Tukey* tests to investigate specific contrasts in the interaction of assessment time – baseline (1), posttest (2), FU3 (3), FU6 (4) – versus intervention groups – NETfacts and NET only.

→ full GLMM is provided in "DatasetS2.xlsx"

SI7: Stigmatization [H4]

Stigmatization was measured with the *Attitudes and Beliefs towards Survivors of Sexual Violence* scale (ATSS; [3]). Each item was rated on a 5 point Likert scale but then dichotomized.

Items 1-4:

1. Strongly disagree that survivors of sexual violence have gotten what they deserve when they get stigmatized and/or socially excluded from the community.
2. Strongly disagree that if I found out that one of my family members were a survivor of sexual violence, I would want it to remain a secret.
3. Strongly disagree that survivors of sexual violence should feel ashamed for what they have done.
4. Strongly agree that I would be willing to care for a family member if s/he was experiencing trouble as a result of sexual violence.

Table SI7_item1. Model details of stigmatization item 1.

Statistical model outputs, showing model fit indices, effect estimates and stigmatization tests for the analysis of the first item ("Strongly disagree that survivors of sexual violence have gotten what they deserve when they get stigmatized and/or socially excluded from the community."), followed by *post hoc Tukey* tests to investigate specific contrasts in the interaction of assessment times – baseline (1), posttest (2), FU3 (3), FU6 (4) – versus intervention groups – NETfacts and NET only.

→ provided in "DatasetS3.xlsx"

Table SI7_item2. Model details of stigmatization item 2.

Statistical model outputs, showing model fit indices, effect estimates and significance tests for the analysis of the first item ("Strongly disagree that if I found out that one of my family members were a survivor of sexual violence, I would want it to remain a secret."), followed by *post hoc Tukey* tests to investigate specific contrasts in the interaction of assessment times – baseline (1), posttest (2), FU3 (3), FU6 (4) – versus intervention groups – NETfacts and NET only.

→ provided in "DatasetS3.xlsx"

Table SI7_item3. Model details of stigmatization item 3.

Statistical model outputs, showing model fit indices, effect estimates and stigmatization tests for the analysis of the first item ("Strongly disagree that survivors of sexual violence should feel ashamed for what they have done."), followed by *post hoc Tukey* tests to investigate specific

contrasts in the interaction of assessment times – baseline (1), posttest (2), FU3 (3), FU6 (4) – versus intervention groups – NETfacts and NET only.

→ provided in "DatasetS3.xlsx"

Table SI7_item4. Model details of stigmatization item 4.

Statistical model outputs, showing model fit indices, effect estimates and stigmatization tests for the analysis of the first item ("Strongly agree that I would be willing to care for a family member if s/he was experiencing trouble as a result of sexual violence."), followed by *post hoc Tukey* tests to investigate specific contrasts in the interaction of assessment times – baseline (1), posttest (2), FU3 (3), FU6 (4) – versus intervention groups – NETfacts and NET only.

→ provided in "DatasetS3.xlsx"

SI8: Skepticism against the reintegration of former combatants [H5]

Table S8. Model details of skepticism against the reintegration of former combatants.

Statistical model outputs, showing model fit indices, effect estimates and significance tests followed by *post hoc Tukey* tests to investigate specific contrasts in the interaction of assessment times – baseline (1), posttest (2), FU3 (3), FU6 (4) – versus intervention groups – NETfacts and NET only.

→ provided in "DatasetS4.xlsx"

SI9: Path models [H6]

→ provided in "DatasetS5.xlsx" for IRMA
→ provided in "DatasetS6.xlsx" for ATSS
→ provided in "DatasetS7.xlsx" for SORS

SI10: Qualitative results extended

The qualitative analysis identified three broad themes reflecting the most significant change of NETfacts health system: 1) Mental Health Improvement; 2) Feelings of belonging; 3) Peace in Everyday Life (See description Table 1). A comparative analysis between the NETfacts and NET only condition was made. The main representation of each group is broadly presented in Figure 1. There were neither clearly contradictory evidence nor inconsistencies in the analyzed data. Most of the themes includes 3 to 5 topics and have different interactions at individual and collective level, and at the NETfacts and NET only condition. These themes are a combination of factual events and processes in lives of the participants and their cognitive interpretation. The only observable difference in factual events are related to consistency and frequency of individual and collective level experiences between the NETfacts and the NET only condition (Figure 1). Most of the topics were more frequently mentioned by the NETfacts communities, except the topic "Economic functioning of former combatants" (topic from theme 3), equally reported by both groups (NETfacts 1).

Table S10a. Definition of identified themes of the most significant change.

**THEMES / Most significant change**	**Description**
**Mental health / individual resilience**	
Intervention NET/NETfacts causal to improvement	*Describes participants' perception of the impact of the therapy (NET and NETfacts) at an individual level, linking counseling, listening and guidance as causal to a reduction of intrusive memories and a reduction of hyperarousal, due to the experience of traumatic events.*
Reduction of intrusive memories	
Reduction of hyperarousal	
**Feelings of belonging**	
Feeling part of the community	*Describes the feeling of being part of the community, with a gradient sense of union among community members. This feeling of belonging and increase in reconciliation affected directly two groups: rape survivors and former combatants. Therapy helped increase community acceptance, normalization and empathy towards these two groups and, therefore, a perceived decrease of stigma and discrimination towards rape survivors and a higher readiness to forgiveness towards former combatants was observed.*
Perception of rape survivors	
Higher readiness to reconstruct and reintegrate former combatants	
**Reduction of conflict in everyday life**	
Better impulse control	*Describes the impact of an increased integration of ex-combatants in the community relationships. Therapy provided a few coping strategies for former combatants, mostly helping them to achieve a better control of their aggressive impulses. Being able to control their aggressiveness towards others had repercussions at different levels: a reduction of*
Reducing criminality in the community	
Increase of feelings of safety in the community	
Economic functioning of former combatants	

Children playing together	*criminality and violent behaviours lead to an increased feeling of safety in the community space; consequently, this personal resilience to better control violent impulses build some trust between community members and ex-combatants creating more interactions and new working opportunities - which allowed former combatants to be more functional economically, by for example, paying their debts.*
Advice others to reduce violence or destructive behaviors	
Increased support and sharing of goods	
Assistance of survivors of sexual violence to receive adequate health care	

SI11: Pre-survey

Since there was no reliable census data, a pre-survey was conducted to assess the number of households and adult inhabitants per village including frequencies for sex and age groups (female vs male, 16 to 36 yrs vs 37 to 57 yrs vs over 57 yrs). For each community, a sample size representative in sex and age was calculated based on the obtained estimates, including a 5% margin of error and a 95% confidence interval (see Table SI11). This estimation was then used as starting point for sampling of participants within the villages.

Table SI11. Pre-survey for census data of adult population representative in sex and age groups.

	Kihindo[1]*	Ngumba2[1]	Maoma[2]	Bukobati[2]*	Kairenge[3]	Katolo*[3]	Total
Number of households	97	148	170	114	64	94	687
Adult population							
Male							
16-36yrs	101	135	145	114	72	75	642
37-57yrs	35	39	57	27	15	33	206
>57yrs	23	30	24	10	20	17	124
Total	159	204	226	151	107	125	972
Female							
16-36yrs	101	123	116	116	79	71	606
37-57yrs	36	44	55	37	31	29	232
>57yrs	30	33	23	20	15	15	136
Total	167	200	194	173	125	115	974
Total	326	404	420	324	232	240	1946

Note. * intervention villages; [1, 2, 3] village pair 1, 2 and 3 with pair 1 and 2 located in North Kivu, pair 3 in South Kivu

References

1. Jacobson, N.S. and P. Truax, *Clinical significance: a statistical approach to defining meaningful change in psychotherapy research.* 1992.
2. Evans, C., F. Margison, and M. Barkham, *The contribution of reliable and clinically significant change methods to evidence-based mental health.* Evidence-Based Mental Health, 1998. **1**(3): p. 70-72.
3. Ferdowsian, H., et al., *Attitudes toward sexual violence survivors: differences across professional sectors in Kenya and the Democratic Republic of the Congo.* Journal of interpersonal violence, 2018. **33**(24): p. 3732-3748.
